# Pediatric diamond-blackfan anemia after hematopoietic stem cell transplantation complicated by bronchiolitis obliterans and air-leak syndrome leading to lung transplantation: a case report with multimodal follow-up

**DOI:** 10.3389/fimmu.2026.1782188

**Published:** 2026-04-22

**Authors:** Wenhui Zhang, Yanxia Zhao, Nan Ge, Feng Hou, Xuewei Li, Chunting Zhao, Hairong Fei, Xue Shi, Wei Wang, Shu Wang, Xiaodan Liu

**Affiliations:** 1 Department of Hematology, the Affiliated Hospital of Qingdao University, Qingdao University, Qingdao, Shandong, China; 2 Department of Bone Marrow Transplantation, The Affiliated Hospital of Qingdao University, Qingdao University, Qingdao, Shandong, China; 3 Children’s Medical Center, Department of Pediatric Hematology and Oncology, The Affiliated Hospital of Qingdao University, Qingdao University, Qingdao, Shandong, China; 4 Department of Lung Transplantation, the Affiliated Hospital of Qingdao University, Qingdao University, Qingdao, Shandong, China; 5 Department of Pathology, The Affiliated Hospital of Qingdao University, Qingdao University, Qingdao, Shandong, China

**Keywords:** air-leak syndrome, bronchiolitis obliterans syndrome, hematopoietic stem cell transplantation, lung transplantation, pediatric

## Abstract

**Introduction:**

Bronchiolitis obliterans syndrome (BOS) is a severe, often fatal pulmonary manifestation of chronic graft-versus-host disease (cGVHD) following allogeneic hematopoietic stem cell transplantation (HSCT). Its progression to air-leak syndrome (ALS) signifies a critical deterioration with exceedingly high mortality. Lung transplantation (LTx) remains a rare salvage option, especially in children, with scarce reports of successful outcomes in those with this complication cascade.

**Case presentation:**

We report the case of a 7-year-old girl with Diamond-Blackfan anemia (DBA) who developed BOS complicated by ALS after allo-HSCT. She developed acute GVHD involving the skin and liver on +100d, which improved following immunosuppressive therapy. On +231d, pulmonary function tests showed severe mixed ventilatory dysfunction (FEV_1_ 37% of predicted value, FEV_1_/FVC 52%), and high-resolution computed tomography (HRCT) revealed mosaic perfusion and bronchial wall thickening, contributing to the diagnosis of BOS. Despite intensive immunosuppressive therapy, she developed ALS on +360d and type II respiratory failure on +475d. Sequential bilateral LTx was performed on October 28, 2025. Postoperatively, the patient recovered following the management of multidrug-resistant bacterial infections and respiratory complications, with no rejection or recurrence of cGVHD during follow-up.

**Conclusion:**

This report presents the youngest documented DBA case of successful LTx for BOS complicated by ALS after allo-HSCT globally. It demonstrates that dynamic multimodal monitoring is crucial for early BOS detection. LTx is a viable therapy for end-stage pulmonary cGVHD in children. This case underscores the need for proactive monitoring in high-risk patients and provides a paradigm for managing this complex complication.

## 1 Introduction

Hematopoietic stem cell transplantation (HSCT) is performed in pediatric patients to cure a range of malignant and non-malignant diseases (1). Diamond-Blackfan anemia (DBA) is a rare genetic disorder caused by mutations in ribosomal protein structural genes, leading to abnormal ribosome biogenesis (2). It is characterized by congenital bone marrow failure syndrome with isolated erythroid hypoplasia and HSCT is the only curative approach (2).

However, chronic graft-versus-host disease (cGVHD), a late complication, remains a major limitation to long-term survival and quality of life (1, 3). Among the diverse manifestations of cGVHD, bronchiolitis obliterans syndrome (BOS) represents a particularly severe pulmonary complication, characterized by progressive and largely irreversible airflow obstruction (4). Studies indicate that the incidence of BOS in children after HSCT is 4.5–8.3% (5, 6). However, due to the smaller diameter of children’s airways and the more fragile mucosal barrier function, the disease progresses more rapidly, and the 5-year survival rate is only 45% to 59% (7).

The clinical course of BOS can be markedly exacerbated by the development of air-leak syndrome (ALS). ALS is caused by the “Macklin effect”, starting with increased intra-alveolar pressure, leading to alveolar rupture, air peeling along the bronchovascular sheath, and the spread of interstitial emphysema to the mediastinum (8). The cumulative incidence of ALS following allo-HSCT is estimated to be between 0.83% and 2.3% (9–11). The onset of ALS is frequently associated with respiratory failure, with mortality rates reported to exceed 60%, resulting in extremely rare cases of successful clinical management (12, 13).

For patients with BOS refractory to intensified immunosuppression, lung transplantation (LTx) remains the only potentially curative option for end-stage lung disease (14). While experience with LTx for end-stage BOS after allo-HSCT has gradually accumulated in adults, the pediatric experience is limited (15). Reports of successful transplantation in children, particularly those with ALS as a high-risk comorbidity, are exceptionally rare.

We report a 7-year-old girl with DBA who developed BOS combined with ALS following allo-HSCT and successfully underwent bilateral LTx. To our knowledge, this is the youngest and most comprehensively documented case of its kind reported globally. Through dynamic multimodal monitoring and literature comparison, we analyze the pathophysiological mechanisms of disease progression and optimizing the diagnosis and treatment decisions based on dynamic multimodal monitoring results, new theoretical basis and practical paradigms can be provided for the precise diagnosis and treatment of complex pulmonary complications after HSCT in pediatrics.

## 2 Case presentation

**Figure 1:**
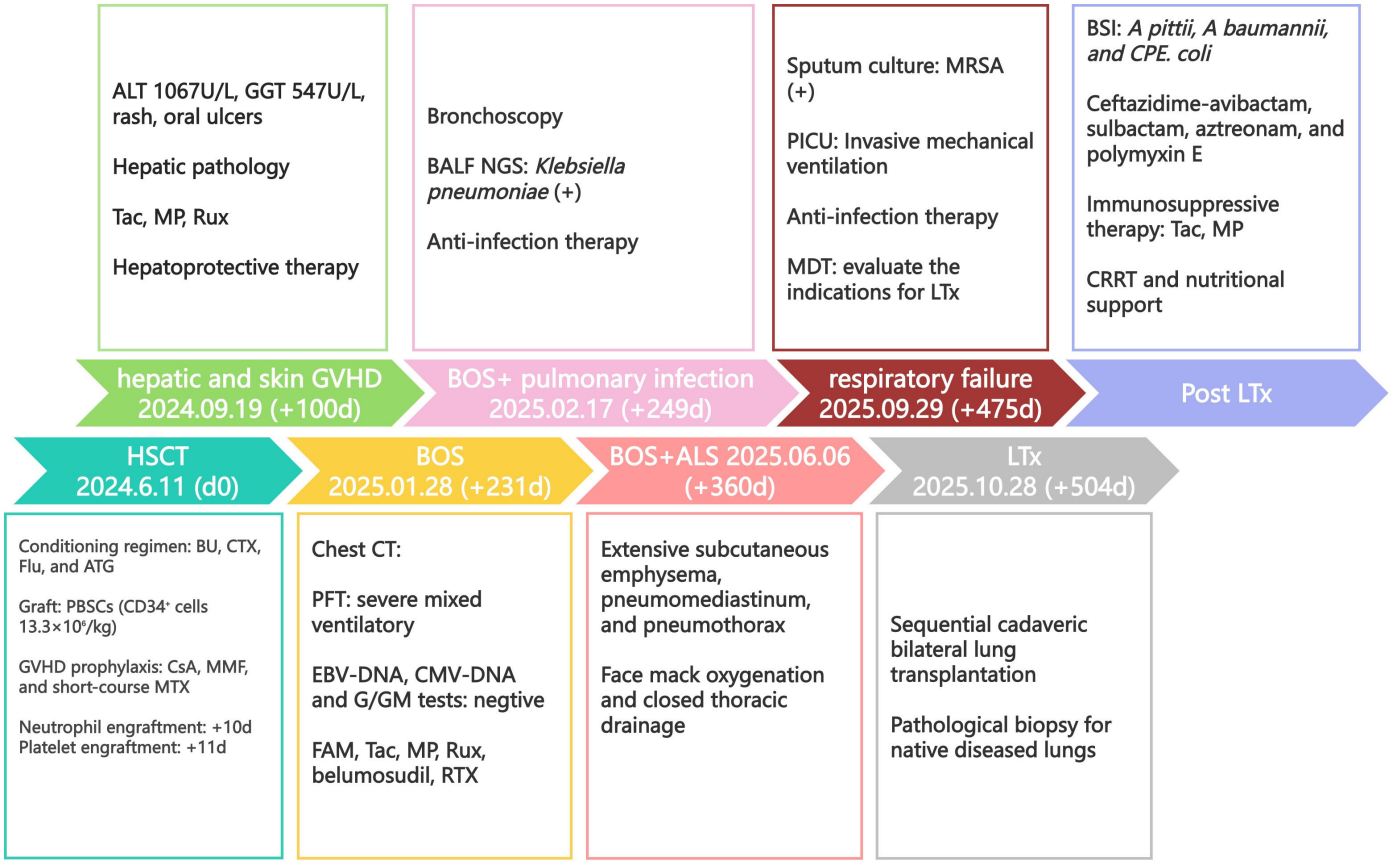
Clinical timeline. A schematic flowchart illustrating the key milestones in the patient’s clinical course, from initial diagnosis and HSCT to the development of BOS, ALS, LTx, and post- LTx milestones. HSCT, hematopoietic stem cell transplantation; GVHD, graft-versus-host disease; ALT, alanine transaminase; GGT, gamma-glutamyl transferase; Tac, tacrolimus; MP, methylprednisolone; Rux, ruxolitinib; BOS, bronchiolitis obliterans syndrome; BALF, bronchoalveolar lavage fluid; NGS, next-generation sequencing; PICU, pediatric intensive care unit; MDT, multidisciplinary team; LTx, lung transplantation; BSI, bloodstream infection; CPE. Coli, carbapenemase-producing Escherichia coli; CRRT, continuous renal replacement therapy; BU, busulfan; CTX, cyclophosphamide; Flu, fludarabine; ATG, antithymocyte globulin; PBSCs, peripheral blood stem cells; CsA, cyclosporine A; MMF, mycophenolate mofetil; MTX, methotrexate; PFTs, pulmonary function tests; EBV, Epstein-Barr virus; CMV, cytomegalovirus; G/GM = galactomannan; FAM, budesonide/formoterol + azithromycin + montelukast; RTX, rituximab; ALS, air-leak syndrome.

**Figure 2:**
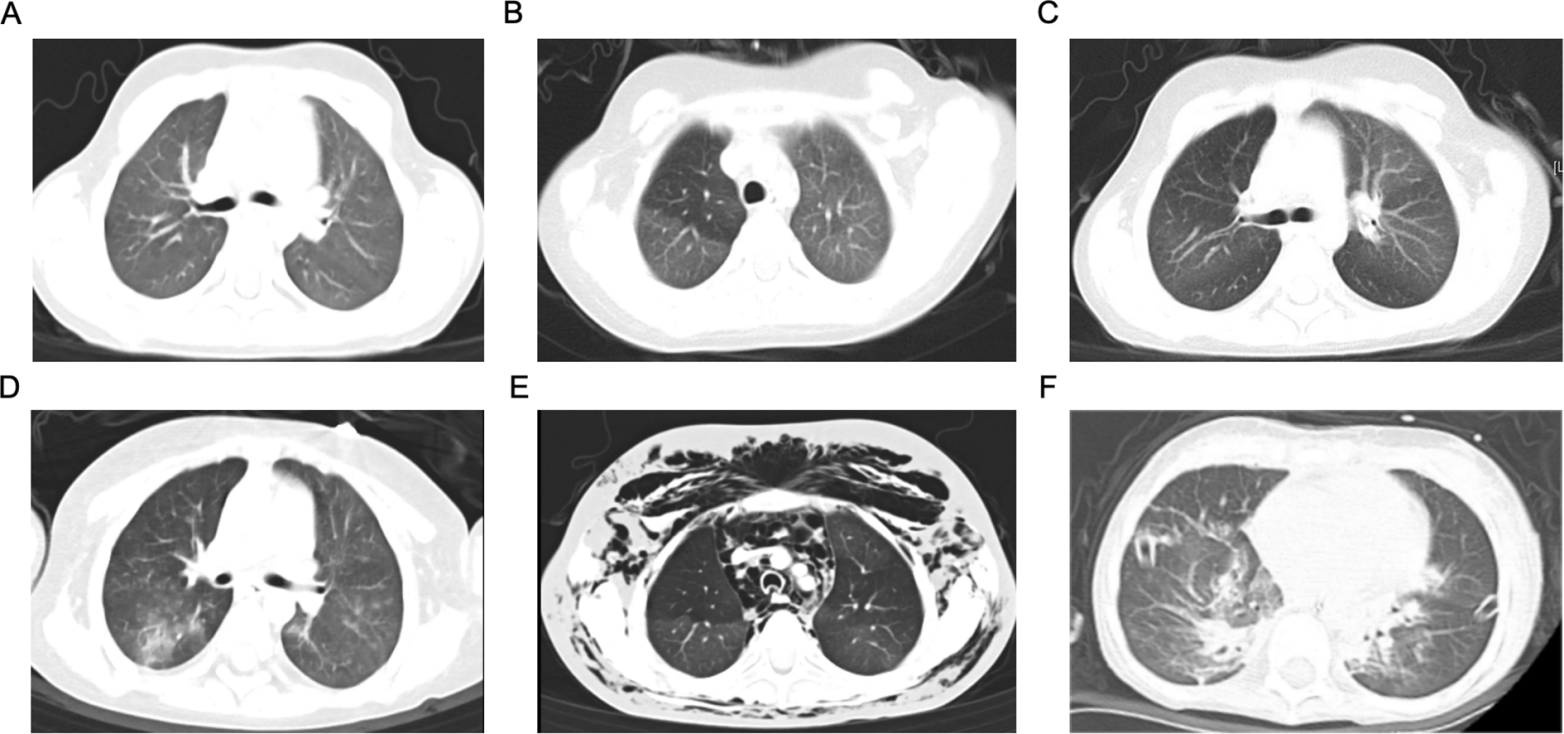
Chest CT findings of HSCT. **(A)** Axial chest CT prior to HSCT showing unremarkable lung parenchyma. **(B, C)** Axial chest CT during BOS episode post-HSCT demonstrating mosaic perfusion pattern **(B)** and bronchial wall thickening **(C, D)** Axial chest CT during a post-HSCT pulmonary infection episode revealing corresponding infectious infiltrates. **(E)** Axial chest CT after development of ALS, showing bilateral atelectasis, extensive subcutaneous emphysema, pneumomediastinum, and pneumothorax. **(F)** Axial chest CT following lung transplantation. HSCT, hematopoietic stem cell transplantation; BOS, bronchiolitis obliterans syndrome; ALS, air-leak syndrome.

We report a 7-year-old girl with DBA. The patient, diagnosed at one year of age, had developed corticosteroid-refractory disease and remained transfusion-dependent. **Figure 1** illustrates the clinical timeline of the patient until December 2025. Before HSCT, chest CT showed no significant abnormalities (**Figure 2A**). And pulmonary function tests showed normal spirometry values (FEV1/FVC: 87%; FEV1: 114% of predicted). She had no contraindications and had indications for allo-HSCT. She accepted matched unrelated donor allo-HSCT on June 11, 2024 as definitive therapy. The myeloablative conditioning regimen comprised busulfan (4.4mg/kg, -8~-7d), cyclophosphamide (30mg/kg, -5~-2d), fludarabine(30mg/m^2^, -6~-2d), and anti-thymocyte immunoglobulin (2.5mg/kg, -5~-2d). A peripheral blood stem cell graft was administered at a CD34^+^ cell dose of 13.3×10^6^ cells/kg. GVHD prophylaxis consisted of cyclosporine A (2.5mg/kg/day, maintain the blood drug concentration between 150ng/ml and 250ng/ml), mycophenolate mofetil (30mg/kg/day), and short-course methotrexate (15mg/m^2^ on +1d, and 10mg/m^2^ on +3d, +5d, and +11d). Neutrophil and platelet engraftment (neutrophil≥0.5×10^9^/L for 3days and platelet≥20×10^9^/L for 7 days without infusion) were documented on +10d and +11d, respectively. At +24d, the donor cell chimerism rate was complete donor chimerism for 99.71%.

On +100d (September 19, 2024), the patient developed rash, oral ulcer, abnormal liver function, and hepatomegaly without pain and ascitic fluid. Subsequently, a liver biopsy was performed, and the pathology indicated mild proliferation of fibrous tissue in the portal areas, with a few lymphocytes infiltrating. The epithelium of the small bile ducts in the portal areas showed degeneration, and no signs of venulitis in the portal areas or central venous inflammation were observed. The patient was diagnosed as skin and liver acute GVHD (aGVHD) and was treated with methylprednisolone (2mg/kg/day), tacrolimus (0.03mg/kg/day, maintain the blood drug concentration between 7 and 12 ug/L), ruxolitinib (10mg/day), mycophenolate mofetil (0.5g/day), and specific liver protective measures. After treatment, her clinical condition improved significantly: alanine transaminase (ALT), aspartate transaminase (AST), and bilirubin levels normalized, while cutaneous rashes and oral ulcers completely resolved. The methylprednisolone dose was gradually tapered off until discontinuation, ruxolitinib was reduced to 2.5 mg twice daily, while tacrolimus and mycophenolate mofetil were maintained.

**Figure 3:**
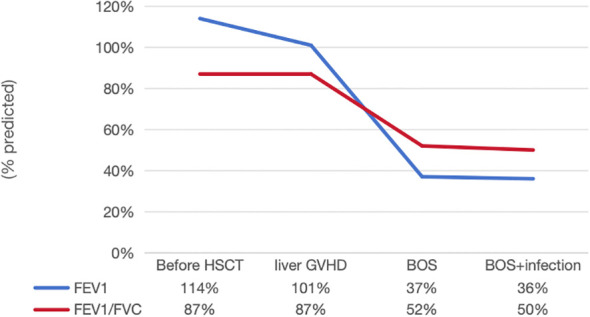
Pulmonary function tests over time. Spirometry trends demonstrating the progressive decline in lung function. Forced expiratory volume in 1 second (FEV_1_) and the FEV_1_/forced vital capacity (FVC) ratio are plotted against key clinical time points (pre-HSCT, liver GVHD, BOS diagnosis, BOS with infection, and pre-transplantation). The sharp decline coinciding with BOS diagnosis and the subsequent further drop are highlighted. FEV1, forced expiratory volume in 1 second; FVC, forced vital capacity; HSCT, hematopoietic stem cell transplantation; GVHD, graft-versus-host disease; BOS, bronchiolitis obliterans syndrome.

On +231d (January 28, 2025), the patient developed progressive exertional dyspnea and wheezing. EBV-DNA, CMV-DNA, and β-D-glucan/galactomannan (G/GM) were tested to be negative, and sputum culture showed no growth of pathogenic bacteria. Pulmonary function tests indicated severe mixed ventilatory dysfunction (**Figure 3**: FEV1/FVC 52%, FEV1 at 37% of predicted value). High-resolution CT showed a mosaic perfusion pattern and bronchial wall thickening (**Figures 2B, C**). She was diagnosed with BOS and was treated with the FAM regimen including inhaled budesonide (3mg/day), azithromycin (5mg/kg/day for 3 consecutive days each week), and montelukast (5mg/day), as well as methylprednisolone (0.5mg/kg/day), tacrolimus (1mg/day), ruxolitinib (5mg/day), belumosudil (0.1g/day), and rituximab (100mg/week for 4 weeks).

On +249d (February 17, 2025), the patient developed a pulmonary infection on the basis of BOS (**Figure 2D**). Bronchoscopy showed bronchial mucosal inflammation, extensive bubbly fluid exudation, and partial sputum obstruction in the right lower lobe (**Supplementary Figures 1A–D**). Next-generation sequencing (NGS) of bronchoalveolar lavage fluid (BALF) indicated Klebsiella pneumoniae infection, and the patient improved after receiving antibiotic treatment.

On day +360 (June 6, 2025), the patient developed mixed ALS. On physical examination, the respiratory rate was 45 breaths/min, which was significantly elevated. Extensive subcutaneous emphysema was observed on the face, neck, and chest, accompanied by a snowball sensation on palpation. On auscultation of the chest, bilateral breath sounds were diminished. HRCT showed bilateral atelectasis, extensive subcutaneous emphysema, mediastinal emphysema, and pneumothorax (**Figure 2E**). The patient was given oxygen inhalation via face mask and closed thoracic drainage, and continued to receive methylprednisolone (0.5mg/kg/day), tacrolimus (1mg/day), and ruxolitinib (5mg/day) for anti-GVHD therapy combined with anti-infection treatment.

On day +475 (September 29, 2025), the ALS were partial resolved after drainage; however, the pulmonary function deteriorated progressively. The patient was transferred to the pediatric intensive care unit (PICU) due to type II respiratory failure and received invasive mechanical ventilation. Blood gas analysis at the time of respiratory failure showed: pH 6.88, PaO_2–_68 mmHg, PaCO_2–_150 mmHg, lactate 1 mmol/L, HCO_3_^-^ unmeasurable. Mechanical ventilation was initiated with pressure-controlled ventilation (PCV) mode: PIP 9 cmH_2_O, PEEP 3 cmH_2_O, respiratory rate 30 breaths/min, FiO_2_ 0.60 (titrated to maintain SpO_2_ > 92%).

A multidisciplinary team (MDT), consisting of specialists from the Department of Hematology, Pediatrics, Respiratory Medicine, Lung Transplantation, and Pediatric Critical Care Medicine, conducted a consultation and evaluated that the patient met the indications for LTx.

After a 3-month waiting period for a suitable lung donor, the patient underwent sequential bilateral LTx under general anesthesia on October 28, 2025 (+504 days post-HSCT). The predicted total lung capacity (pTLC) was calculated using the validated formula for preadolescent children, yielding a donor-to-recipient pTLC ratio of 1.84 (donor height 138 cm; recipient height 109 cm) (16).

**Figure 4:**
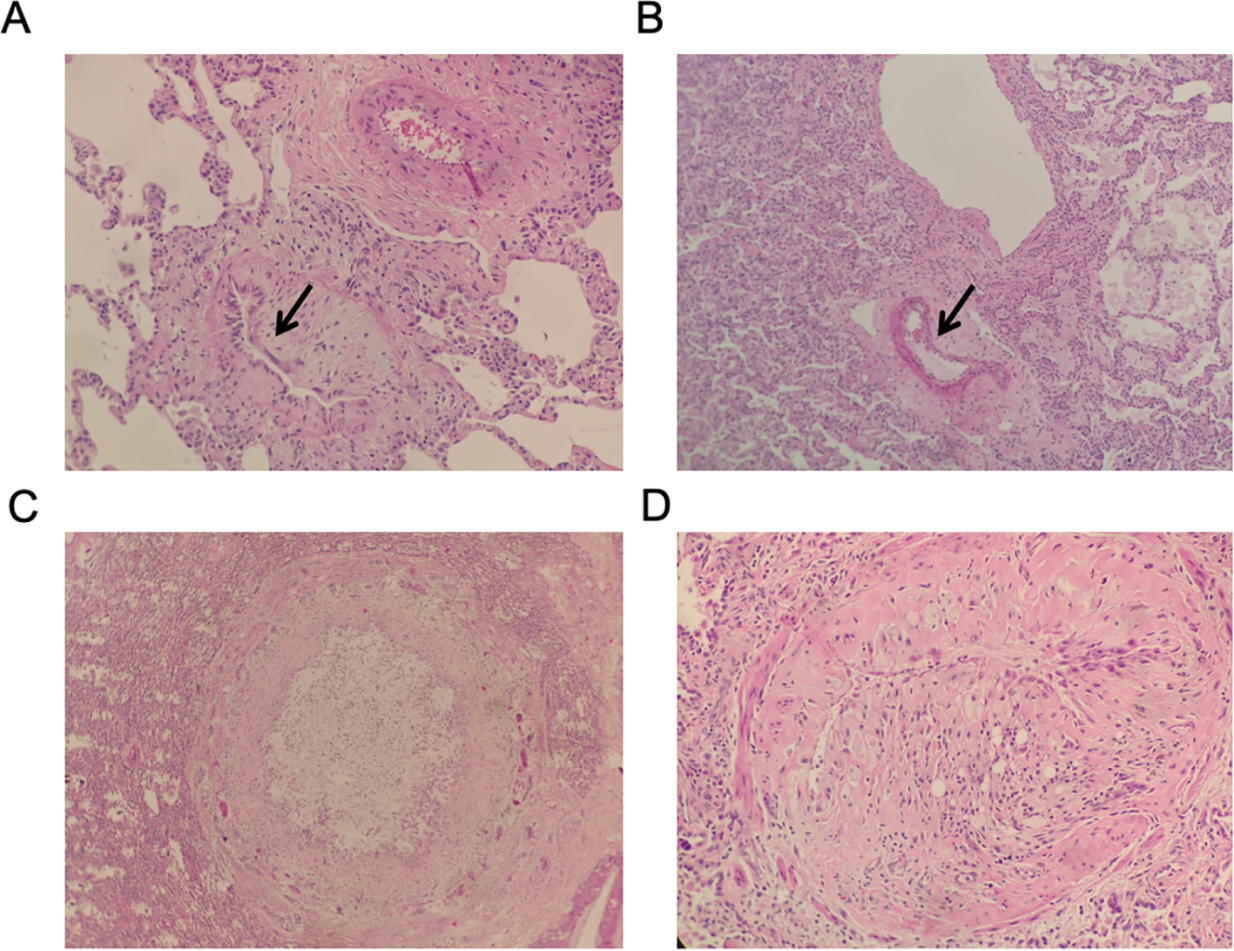
Histopathological findings of the diseased lung tissue in post-HSCT BOS (hematoxylin and eosin [HE] staining). **(A)** Bronchiolar luminal narrowing due to intimal fibrosis (HE staining, ×100). **(B)** Bronchiolar luminal narrowing due to adventitial fibrosis (HE staining, ×100). **(C, D)** Vasculitis characterized by vascular wall thickening and chronic inflammatory cell infiltration (HE staining, ×200). These pathological features confirm the diagnosis of advanced bronchiolitis obliterans syndrome secondary to chronic graft-versus-host disease.

Pathological biopsy of the lesional lung tissue was performed, and the results showed as followings: atrophy of bronchiolar epithelium, proliferation of collagenous fibrous tissue in the bronchiolar wall with luminal stenosis or occlusion; focal consolidation of lung parenchyma, diffuse widening of alveolar septa with massive proliferation of fibroblasts and fibrocytes accompanied by myxoid degeneration, aggregation of foam cells in the alveolar lumina, and proliferation of interstitial fibrous tissue; thickening of the walls of scattered blood vessels with chronic inflammatory cell infiltration, consistent with vasculitic changes. These findings were consistent with BO after HSCT (**Figure 4**).

In the early postoperative period, the patient received mechanical ventilation support via orotracheal intubation and was successfully extubated on the 4th postoperative day. However, reintubation was performed on the 6th postoperative day due to respiratory muscle weakness, hypercapnia, and complicated airway hemorrhage. Post-reintubation mechanical ventilation parameters: PCV mode with PIP 17 cmH_2_O, PEEP 5 cmH_2_O, respiratory rate 17 breaths/min, FiO_2_ 0.55. Postoperatively, the patient developed multidrug-resistant bacterial bloodstream infection, etiological testing identified Acinetobacter pittii, Acinetobacter baumannii, and carbapenemase-producing Escherichia coli. The anti-infection treatment regimen was adjusted to ceftazidime-avibactam combined with sulbactam, aztreonam, and colistin E. Meanwhile, anti-GVHD therapy with tacrolimus (0.03mg/kg/day, maintain the blood drug concentration between 7 and 12 ug/L) combined with methylprednisolone (2mg/kg/day) was administered, along with renal replacement therapy and nutritional support. The patient was successfully extubated on the 27th postoperative day and subsequently weaned off oxygen gradually. CT examination showed clear graft morphology without evidence of acute rejection or recurrence of cGVHD (**Figure 2F**).

The patient’s guardian has provided written informed consent for the disclosure and use of their information in the preparation and publication of this case report.

## 3 Discussion

We report a case of a 7-year-old girl who developed refractory BOS combined with ALS after undergoing allo-HSCT for DBA. To our knowledge, this child is the youngest case diagnosed with DBA to successfully undergo LTx after developing these complications following allo-HSCT. This case clearly illustrates the progressive sequence of aGVHD, BOS, ALS, and pulmonary function failure, highlighting the limitations of current treatment options. This case offers valuable clinical insight into the pathophysiological progression of chronic pulmonary complications associated with cGVHD. Furthermore, it establishes a clinically actionable framework for managing this high-risk population.

Early detection of BOS in pediatric post-HSCT populations remains challenging due to nonspecific initial symptoms and the invasiveness of definitive diagnostic procedures. Early symptoms in pediatric patients are often subtle, commonly presenting as shortness of breath and wheezing after activity, which can easily be confused with pulmonary infections, leading to delays in diagnosis. Importantly, symptoms may not only be minimal but can also be completely absent during the developing phase of BOS, making routine screening even more critical (8, 17). Although pathological examination remains the gold standard for the diagnosis of obliterative bronchiolitis, lung biopsy is associated with severe complications including pneumothorax, mediastinal emphysema, persistent air leak syndrome, and even mortality (18, 19).

This case established a multimodal diagnostic framework integrating dynamic pulmonary function monitoring with HRCT that enabled pre-emptive identification of BOS progression and timely transplantation listing. The core value of dynamic pulmonary function monitoring lies in its ability to provide early warnings based on trend analysis. Prior to HSCT and during the liver GVHD phase, both FEV_1_ and the FEV_1_/FVC ratio remained relatively preserved: FEV_1_ stayed above the predicted normal range (114% pre-HSCT, 101% at liver GVHD), while FEV_1_/FVC maintained a stable 87% across these two stages. This stability suggests that early post-HSCT complications targeting the liver exert minimal effect on pulmonary mechanics—a observation consistent with reports that liver GVHD and pulmonary complications post-HSCT involve divergent immune-mediated organ targeting (20). It is worth noting that with the emergence of BOS, both of these indicators experienced a sharp and clinically significant decline: FEV_1_ dropped by approximately 63%, falling to 37% of the predicted value; FEV_1_/FVC decreased by about 40%, dropping to 52% of the predicted value. This sharp reduction directly reflects the pathological hallmark of BOS which impairs expiratory airflow and disrupts ventilatory efficiency (21). A subsequent mild further reduction (to 36% for FEV_1_ and 50% for FEV_1_/FVC) in the phrase of BOS combined with infection underscores the synergistic deleterious effect of secondary infections on pre-existing BOS-related airway damage. According to the 2024 ATS guidelines for the diagnosis of BOS after HSCT in children, a “FEV_1_ decline rate > 15%” can be used as a warning indicator for BOS (22). This threshold prompted immediate intervention in our patient despite absolute values not yet meeting formal BOS criteria. The monitoring results of this case verified the clinical value of this warning indicator, suggesting that for children at high risk of cGVHD, dynamic monitoring of the trend of lung function changes is more diagnostically significant than a single measurement.

Studies have shown that the diagnostic sensitivity of HRCT for BOS in children is 85% to 90% (23). Before HSCT, the HRCT of the children showed no significant abnormalities, but typical mosaic perfusion signs and bronchial wall thickening appeared in the BOS stage, which were characteristic imaging manifestations of BOS (24). When the disease progresses to the ALS stage, HRCT further reveals atelectasis, extensive subcutaneous emphysema, and mediastinal emphysema, thereby visually reflecting the air leakage caused by the destruction of the airway structure. The combined application of HRCT and pulmonary function monitoring in this case successfully avoided diagnostic delays and gained time for early intervention. Importantly, while multimodal monitoring offers a non-invasive diagnostic basis, pathological confirmation remains indispensable for clarifying disease severity and the underlying progression mechanisms—a notion corroborated by pathological examinations of the explanted lung tissue in the present case. Based on these observations, we recommend routine dynamic screening with HRCT and pulmonary function tests for high-risk children with GVHD after allo-HSCT, especially when unexplained respiratory symptoms occur, to avoid missed diagnosis or misdiagnosis.

The pathological results of the explanted lungs validated the accuracy of the preoperative diagnosis of BOS and played a key role in understanding how severe and fast-progressing the disease was. The definitive features of obliterative bronchiolitis included bronchiolar luminal stenosis or occlusion caused by submucosal and adventitial fibrosis. These changes explained the irreversible airflow obstruction observed in pulmonary function tests. The concurrent vasculitis, characterized by vascular wall thickening and chronic inflammatory cell infiltration, not only compromised pulmonary microcirculation—potentially worsening epithelial ischemia and fibrosis—but also likely reduced the delivery of immunosuppressive agents to target tissues, leading to treatment resistance (25). Furthermore, inflammation damaged the bronchovascular sheath, which may have allowed air to leak, causing the extensive subcutaneous and mediastinal emphysema typical of ALS (26). The multi-dimensional confirmation of HRCT, pulmonary function, and pathology has established a complete evidence chain of “morphology-function-pathology,” providing support for analyzing disease progression mechanisms and optimizing diagnosis and treatment decisions.

This extensive lesion, involving the airway, vascular, and interstitial regions, explains the rapid progression of BOS to ALS observed in this case. Children’s unique anatomical vulnerabilities—smaller airway diameters causing disproportionately greater obstruction from equivalent inflammation or fibrosis, incompletely developed elastic fibers, and fragile alveolar walls—amplify these pathological effects and account for the significantly higher ALS risk in pediatric BOS compared to adults (27). Given this accelerated disease trajectory and the extremely poor prognosis of BOS, minimizing modifiable risk factors should be a priority in pretransplant planning, especially for non-malignant diseases like DBA where the risk-benefit balance differs from malignancies. Key preventive strategies include: conditioning regimen optimization, where reduced-intensity conditioning (RIC) protocols may offer lower pulmonary toxicity compared to busulfan-based myeloablative regimens—although this benefit must be carefully weighed against the increased risk of graft rejection, particularly in the setting of unrelated donor transplantation (28); GVHD prophylaxis incorporating post-transplant cyclophosphamide (PTCy), which has recently been associated with a reduced incidence of BOS (29); graft source selection, with bone marrow potentially conferring a lower risk of BOS compared to peripheral blood stem cells (30, 31); and respiratory virus prevention strategies, including vaccination administered after immune reconstitution and anti-infection prophylaxis (32). This case illustrates accelerated disease progression potentially driven by both the busulfan-based myeloablative conditioning and pulmonary infections complicating post-transplant BOS (33, 34). Despite GVHD-prophylactic measures—including shortening busulfan exposure to 2 days and administering prophylactic acyclovir and posaconazole following transplantation—GVHD still developed in this patient. This interplay between pathological vulnerabilities and environmental exposures underscores the critical importance of pretransplant risk factor modification and early intervention in high-risk pediatric patients.

Glucocorticoids remain the first−line therapy for BOS after HSCT, yet corticosteroid use itself constitutes a critical risk factor for persistent air leak and pneumothorax (35, 36). The pathophysiological basis for this paradox lies in corticosteroid-mediated impairment of wound healing and tissue repair mechanisms. Corticosteroids delay alveolar epithelial regeneration, impair collagen synthesis, and reduce tensile strength of healing tissues (37). In the context of pre-existing lung injury, these effects may transform manageable air leaks into persistent, life-threatening complications. Future therapeutic strategies must address this paradox through targeted approaches that preserve immunosuppressive efficacy while minimizing pulmonary toxicity. The development of steroid-sparing regimens, including JAK inhibitors, tyrosine kinase inhibitors, and extracorporeal photopheresis, offers potential pathways to circumvent this dilemma (38). When medical therapy fails, as in this case with refractory BOS complicated by ALS and type II respiratory failure, LTx represents the only curative option (39).

Post-HSCT recipients present unique selection considerations that influenced our MDT decision-making. Firstly, a stable donor chimerism, especially complete chimerism, is associated with a reduced risk of GVHD and the establishment of immune tolerance (40). Preclinical models have demonstrated that stable donor chimerism correlates with improved acceptance of lung allografts in combined lung and bone marrow transplantation (40). Secondly, several studies have demonstrated that HLA mismatch between lung donors and recipients is a well−established adverse prognostic factor, associated with inferior survival and an increased risk of chronic lung allograft dysfunction (CLAD) (41, 42). Furthermore, organ size matching poses particular challenges in pediatric LTx. Oversized grafts carry the risk of atelectasis and impaired hemodynamics, whereas undersized grafts may result in residual pleural space and prolonged air leak. In this patient, the prior HSCT was carried out using a fully HLA−matched donor, and complete donor chimerism was confirmed prior to LTx. Following comprehensive evaluation by a MDT and consideration of these favorable factors, the child was expeditiously listed for LTx. Although the donor to recipient pTLC ratio indicated substantial graft oversizing relative to recipient capacity, anatomical compatibility was supported by well-matched thoracic dimensions: chest circumference measured 60 cm in the donor versus 58 cm in the recipient. Consequently, the donor lungs were deemed clinically appropriate for transplantation based on physical dimensional compatibility.

Standard LTx recipients typically receive triple-drug maintenance therapy comprising calcineurin inhibitors (CNIs, such as cyclosporine or tacrolimus), glucocorticoids, and a third agent (antiproliferative agents such as azathioprine or mycophenolate mofetil) (43). In contrast, first-line immunosuppressive therapy for cGVHD following HSCT consists of glucocorticoids with or without CNIs (44). However, no evidence-based guidelines or prospective studies currently exist to inform standardized immunosuppressive management in this unique population with pre-existing cGVHD undergoing LTx. This therapeutic gap necessitates individualized strategies that must achieve precise equilibrium among three competing therapeutic objectives: prevention of lung allograft rejection, control of underlying cGVHD disease activity, and mitigation of substantially elevated infectious risk attributable to overlapping immunosuppression (45). For HSCT recipients with prior CNI-induced nephrotoxicity, a once-daily cyclosporine regimen may be adopted post-LTx, with dose individualization guided by area under the concentration-time curve (AUC) monitoring and lower target trough concentrations (e.g., 100–150 ng/mL during the first postoperative month) to preserve residual renal function (46). In summary, HSCT recipients with cGVHD undergoing LTx require highly individualized immunosuppressive modifications built upon standard LTx protocols, with careful consideration of pre-existing cGVHD organ involvement patterns, prior immunosuppressive drug exposure history, and the dynamic interplay between rejection and infectious risks following LTx (43, 47).

This case report has several limitations. First, diffusing capacity for carbon monoxide (DLco) and static lung volume testing (including total lung capacity [TLC] and residual volume [RV]) were not performed due to technical limitations and patient cooperation issues in this pediatric setting. These parameters could have provided earlier detection of small airway dysfunction and air trapping before the significant FEV1 decline, potentially enabling earlier intervention (48, 49). Second, the single-case nature limits generalizability, and prospective studies are needed to validate the monitoring and treatment strategies described. Third, long-term follow-up after LTx is ongoing, and late complications including chronic lung allograft dysfunction require continued surveillance.

## 4 Conclusion

This report describes the successful sequential bilateral LTx in a 7-year-old girl with DBA who developed refractory BOS complicated by ALS after allo-HSCT. Dynamic multimodal monitoring, including serial pulmonary function tests and HRCT, enables early detection of BOS. Pathological examination clarifies the BOS to ALS progression mechanism related to pediatric-specific vulnerabilities. MDT collaboration and recognizing ALS as a “transplant warning signal” ensure timely curative transplantation. The refractory nature of the disease underscores the need for early intervention in high-risk patients. LTx has proven effective, but long-term follow-up and further research on early warning and prophylactic strategies are needed to improve outcomes of complex post-HSCT pulmonary complications in pediatrics.

## Data Availability

The original contributions presented in the study are included in the article/**Supplementary Material**. Further inquiries can be directed to the corresponding author.
