# Supplementary material for: Pediatric diamond-blackfan anemia after hematopoietic stem cell transplantation complicated by bronchiolitis obliterans and air-leak syndrome leading to lung transplantation: a case report with multimodal follow-up
Source: Front Immunol. 2026 Apr 22;17:1782188. doi: 10.3389/fimmu.2026.1782188 (PMC13143672; doi:10.3389/fimmu.2026.1782188)
Supplement: Supplementary file 1 [file Table1.docx]

Supplementary Figure 1. Bronchoscopic findings during the period of BOS combined with infection.

**(A)** Congestion of the left upper lobe bronchial mucosa.

**(B)** Scattered bubble-like viscous secretions within segmental bronchi.

**(C)** Congestion of the right bronchial mucosa.

**(D)** Marked mucous plugging in multiple branches of the right lower lobe bronchus.

Abbreviations: BOS, bronchiolitis obliterans syndrome.
