# Supplementary figures and images for: Pediatric diamond-blackfan anemia after hematopoietic stem cell transplantation complicated by bronchiolitis obliterans and air-leak syndrome leading to lung transplantation: a case report with multimodal follow-up
Source: Front Immunol. 2026 Apr 22;17:1782188. doi: 10.3389/fimmu.2026.1782188 (PMC13143672; doi:10.3389/fimmu.2026.1782188)

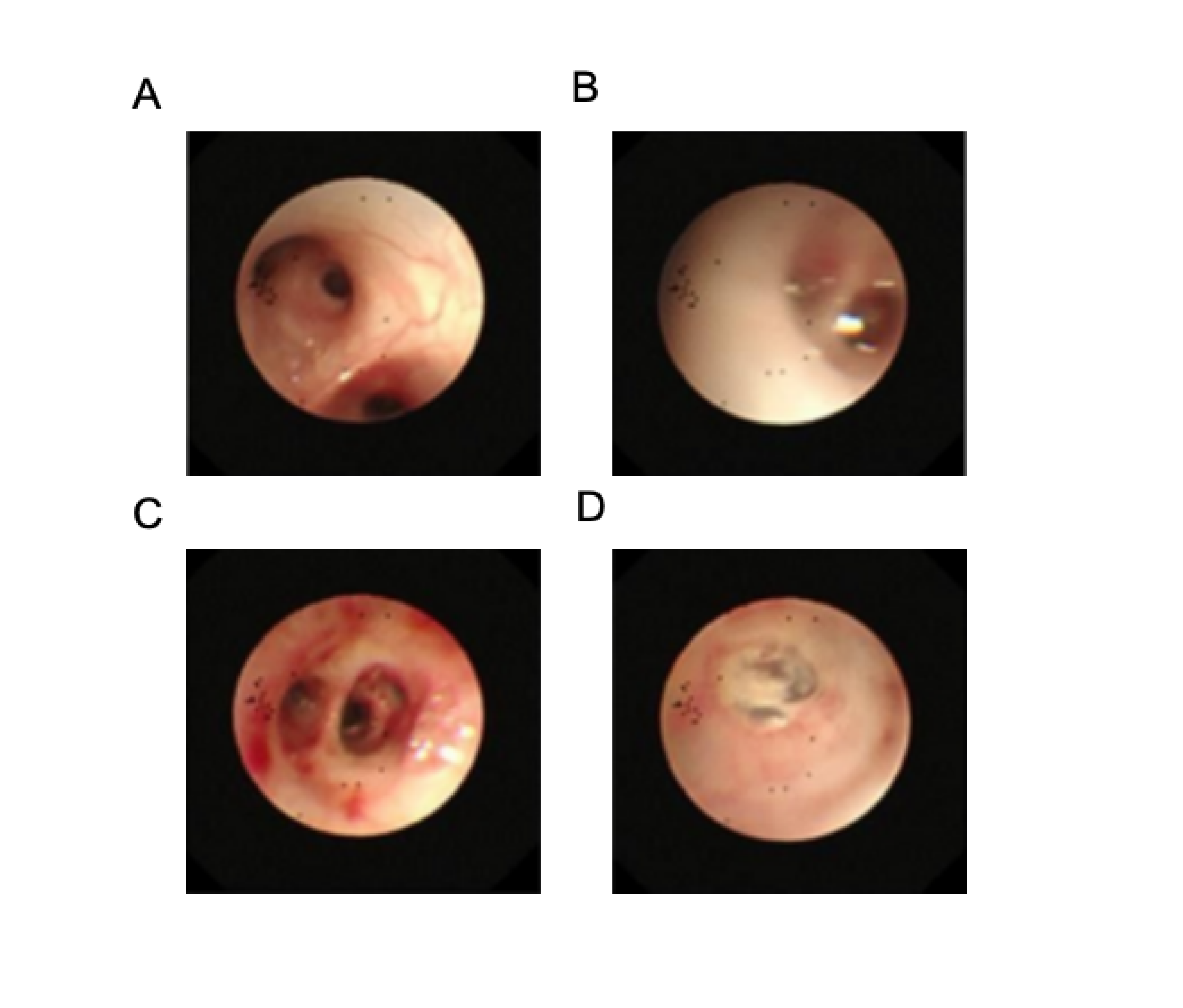

Supplement: Supplementary file 2 [file Image1.tif]
